# Comparison of Different Irrigants in the Removal of Endotoxins and Cultivable Microorganisms from Infected Root Canals

**DOI:** 10.1155/2015/125636

**Published:** 2015-08-09

**Authors:** Marcia Carneiro Valera, Flávia Goulart da Rosa Cardoso, Adriana Chung, Ana Cláudia Carvalho Xavier, Mariana Diehl Figueiredo, Frederico Canato Martinho, Renato Miotto Palo

**Affiliations:** Department of Restorative Dentistry, Institute of Science and Technology, Universidade Estadual Paulista (UNESP), São José dos Campos 12245-000, SP, Brazil

## Abstract

This study was conducted to compare the effectiveness of different irrigants used to remove endotoxins and cultivable microorganisms during endodontic therapy. Forty root canals were contaminated and divided into groups according to the irrigant: 2% NaOCl + surfactant, 2% CHX, 2.5% NaOCl, and pyrogen-free saline solution (control). Samples were collected after root canal contamination (S1), after instrumentation (S2), and 7 days after instrumentation (S3). Microorganisms and endotoxins were recovered from 100% of the contaminated root canals (S1). At S2, 2% NaOCl + surfactant, 2% CHX, and 2.5% NaOCl were able to completely eliminate cultivable microorganisms. At S3, both 2% CHX and 2.5% NaOCl were effective in preventing *C. albicans* and *E. coli* regrowth, but *E. faecalis* was still detected. No microorganism species was recovered from root canals instrumented with 2% NaOCl + surfactant. At S2, a higher percentage value of endotoxin reduction was found for 2% NaOCl + surfactant (99.3%) compared to 2% CHX (98.9%) and 2.5% NaOCl (97.18%) (*p* < 0.05). Moreover, at S3, 2% NaOCl + surfactant (100%) was the most effective irrigant against endotoxins. All irrigants tested were effective in reducing microorganisms and endotoxins from root canals. Moreover, 2% NaOCl + surfactant was the most effective irrigant against endotoxins and regrowth of microorganisms.

## 1. Introduction

Microorganisms and their metabolites are intimately related to the etiology of pulp and periapical pathology. The removal/elimination of pulp remnants, microorganisms, and by-products are important for endodontic treatment success [1, 2].

The polymicrobial nature of endodontic infections is reported in the literature [3, 4].* Enterococcus faecalis* and* Candida albicans* are likely candidates.* Enterococcus faecalis*, a Gram-positive bacterium commonly detected in persistent infections, has the capacity to deeply penetrate into dentine tubules and, thus, endures after use of bactericidal substances during endodontic treatment [5].* Candida albicans* is a versatile yeast, being able to adapt to different levels of pH and capable of proliferating on dentin surface and penetrating into dentin tubules [6].

Lipopolysaccharide (LPS), generally referred to as endotoxin, is present in the outer cell membranes of Gram-negative bacteria and is released during cell division or cell death [7], being one of the most important virulence factors involved in the development and maintenance of periapical inflammation and clinical symptomatology [3, 4, 8, 9]. Due to its toxicity to pulp and periapical tissues, special attention has been given to the complete removal/neutralization of endotoxin from infected root canals [3, 8, 9]. LPS from* Escherichia coli* (a facultative anaerobic straight rod-shaped Gram-negative bacteria), considered standard endotoxins, has been used to test the ability of different irrigants in reducing endotoxin [9].

Root canals with complex anatomy limit the mechanical action of endodontic instruments, and, thus, the use of chemical solutions with antimicrobial activity, ability to dissolve organic tissues, lubricant properties, and low cytotoxicity is highly recommendable as an adjunct to the mechanical preparation [10].

Sodium hypochlorite (NaOCl), the most popular irrigant, shows a potent antimicrobial activity and provides effective dissolution of both necrotic tissues [11] and organic components of the smear layer [12]. However, it is highly irritating to periapical tissues at high concentrations [13, 14]. Chlorhexidine (CHX) is an alternative substance with wide spectrum acting over Gram-positive bacteria, Gram-negative bacteria, and yeasts [15]. Moreover, it remains significantly longer inside the root canals after instrumentation [16] and is less toxic than NaOCl [10]. However, there is concern on the use of CHX, since it is unable to dissolve pulp tissues.

High surface tension could affect the ability of auxiliary chemical substances to penetrate the dentin and thus reduce antibacterial effectiveness within dentinal tubules, which is dependent on their wettability. The wettability of certain solutions relies on the surface tension [17, 18] of ideal surfaces (chemically homogeneous, flat, nonreactive, undeformable, and not swollen by the wetting liquid) and on surface properties of the dentin [19]. The efficiency of an irrigant could be thus improved by reducing its surface tension, consequently, increasing its diffusion into the root canals [20, 21]. The improvement of the wettability of an auxiliary chemical substance may contribute to its capacity to dissolve organic tissues and increase antimicrobial activity [21]. The association of NaOCl with a specific substance aiming at these characteristics and properties (i.e., surfactant) can improve its function.

It is known that using heat or adding chemicals (e.g., surfactants) can reduce the surface tension of an irrigant [17, 18, 21–23]. Surfactant molecules are characterized by a hydrophobic portion, organic/oil soluble or water insoluble, and a hydrophilic region (often polar), water soluble [17, 18, 21–23]. In particular, nonionic agents do not ionize but contain hydrophilic polar groups and/or hydrogen bonding capabilities, which can provide strong interactions with water molecules, improving solubility [24].

Therefore, the aim of the present study was to evaluate in vitro the antimicrobial effect of 2.5% sodium hypochlorite, 2% chlorhexidine solution, and an experimental solution of 2% sodium hypochlorite associated with a surfactant against* Enterococcus faecalis*,* Candida albicans*,* Escherichia coli*, and their endotoxins, within root canals.

## 2. Material and Methods

This study was previously approved by the local human research ethics committee of the São Paulo State University (UNESP), São José dos Campos, Brazil.

Forty single-rooted teeth (incisors, canines, and premolars) were cleaned and immersed in saline solution until use. The selection of the teeth was based on the size and morphological similarity of their roots, and the teeth were then stratified into the groups tested. The crowns were sectioned with carborundum disc to standardize the length of the specimens at 16 ± 0.5 mm. In order to standardize the apical diameter of the teeth selected, the full length of the root canals was instrumented up to a #30 Kerr file (Dentsply Ind. Com. Ltda., Petrópolis, RJ, Brazil), followed by irrigation with 3 mL saline solution after each instrumentation. Next, the canals were filled with EDTA for 3 minutes and irrigated with 10 mL of saline solution. The apical region of each tooth was sealed with light-cured composite resin (Z-100, 3M, Saint Paul, USA) and the roots were externally sealed with 2 layers of epoxy adhesive (Brascola, São Paulo, SP, Brazil), except the cervical opening. The specimens were randomly placed in cell culture plates containing 24 wells (Easypath, São Paulo, SP, Brazil). All culture plates and other materials used in the present study were sterilized by gamma irradiation (Embrarad, Cotia, SP, Brazil) for elimination of preexisting endotoxins.

Initially, a suspension of 10^6^ cells/mL of* E. coli* species (ATCC 25922) was prepared. Next, 10 *μ*L of this* E. coli* suspension was inoculated into each root canal followed by 10 *μ*L of brain heart infusion (BHI) broth (Himedia Laboratories, Mumbai, India). A sterile cotton pellet was soaked in the culture medium and placed in the cervical third of the root canals. All specimens were stored in an incubator at 37° ± 1°C in humidified atmosphere. BHI broth was added to root canals every three days [25]. After 7 days, 5 *μ*L of* C. albicans* suspension (ATCC 18804), 5 *μ*L of* E. faecalis* suspension (ATCC 29212), and 10 *μ*L of BHI broth were added to the root canals prior to storage of all the specimens in incubator at 37° ± 1°C and humidified atmosphere for 21 days. BHI broth was added to fill up completely the root canal lumen every three days.

After verifying the contamination (baseline samples, S1), all teeth were instrumented into their full length up to K-file #50 and then irrigated with 3 mL irrigating solution after each instrumentation by using a total of 12 mL of the irrigating solution for each tooth.

The specimens were divided into three experimental groups (*n* = 10 each) according to the irrigating solution used: GI: 2% sodium hypochlorite + chloride alkali electrolyte-stable anionic surfactant (experimental solution; Ultradent Products, UT, USA); GII: CHX 2% chlorhexidine solution (Ultradent Products, UT, USA); and GIII: 2.5% sodium hypochlorite (Asfer Indústria Química Ltda, São Caetano do Sul, Brazil). The control group (GIV) was irrigated with pyrogen-free saline solution (Aster Produtos Médicos Ltda, Sorocaba, SP, Brazil).

After instrumentation, NaOCl and NaOCl + surfactant were inactivated with 5 mL of sterile 0.5% sodium thiosulfate, whereas CHX was inactivated with 5 mL of a solution containing 5% Tween 80 and 0.07% (w/v) lecithin during 1-minute period, which was removed with 5 mL of sterile/apyrogenic water. In order to determine the antimicrobial activity of the irrigants, a second sampling was performed (S2). To determine the residual antimicrobial activity of the irrigant solutions, the root canals were filled with saline solution and stored in an incubator at 37° ± 1°C for seven days. A final sampling (S3) was performed to determine the residual antimicrobial activity of the irrigants.

All sampling procedures (S1, S2, and S3) were carried out in the same standard way as follows: the root canals were filled with saline solution, and 100 *μ*L of the root canal content was collected and transferred to Eppendorf tubes containing 900 *μ*L of saline solution [9]. For endotoxin quantification, the samples were diluted to 1 : 100 because of the sensitivity of the kinetic chromogenic* limulus amebocyte lysate* test (KQCL).

### 2.1. Determination of Cultivable Bacterial Counts (Culturing Procedure)

To evaluate the antimicrobial activity, aliquots of 100 *μ*L of all samples collected at S1 (baseline samples), S2 (after instrumentation), and S3 (after 7 days of instrumentation) were seeded in Sabouraud dextrose agar (Himedia Laboratories, Mumbai, India) supplemented with chloramphenicol for* Candida albicans*, Enterococcosel agar (Himedia Laboratories, Mumbai, India) for* Enterococcus faecalis*, and MacConkey agar (Himedia Laboratories, Mumbai, India) for* Escherichia coli.* Then, all microorganisms were incubated at 37°C for 24 hours, and the number of colony-forming units (CFU/mL) was counted.

### 2.2. Quantification of Endotoxins (Endotoxins Procedures)

The kinetic chromogenic limulus amebocyte lysate assay (Lonza, Walkersville, MD, USA) was used for quantification of endotoxins.* Escherichia coli* endotoxin was used as standard. For the test, 100 *μ*L of the root canal samples was added to 96-well plate. The samples were run in quadruplicate. Standard curve was performed in order to determine the levels of endotoxins present in the root canal samples, according to the manufacturer instructions. A spike procedure was performed according to the manufacturer's instructions in order to avoid possible interferences of contaminants present in the root canal samples with the recovery of endotoxins by the LAL test. Thus, each sample duplicated in the 96-well apyrogenic plate (Easypath, São Paulo, SP, Brazil) was contaminated with a known concentration of endotoxin (10 EU/mL). The plate was incubated at 37°C ± 1°C for 10 minutes in a Kinetic-QCL reader, which was coupled to a microcomputer with the WinKQCL software. Next, 100 *μ*L of chromogenic reagent was added to each well. After the beginning of the kinetic test, the software continuously monitored absorbance at 405 nm in each microplate well and automatically calculated the log/log linear correlation between reaction time of each standard solution and corresponding endotoxin concentration.

The results were submitted to the Kruskal-Wallis and Dunn tests, with a level of significance at 5%.

## 3. Results

### 3.1. Microbiological Analysis

At the baseline (S1), microorganisms were recovered from 100% of the contaminated root canals (40/40), with a median CFU-count ranging from 10^5^ to 10^7^ CFU/mL. At S2, 2% NaOCl + surfactant, 2% CHX, and 2.5% NaOCl were able to completely eliminate the target microorganisms in most of the root canals analyzed. After instrumentation (S2), no statically significant differences were found by comparing the median percentage reductions in CFU, as shown in Table 1 (*p* > 0.05), except for the saline solution (control group), which showed the lowest effectiveness in reducing the bacterial load (*p* < 0.05) (Table 1). After 7 days of root canal instrumentation (S3), both 2% CHX (GII) and 2.5% NaOCl (GIII) were effective in preventing* C. albicans* and* E. coli* regrowth, but* E. faecalis* was still detected in root canal samples from GII and GIII. No microorganism species was recovered from root canals instrumented with 2% NaOCl + surfactant (GI).

Table 1 shows the median percentage reductions in bacterial load and respective range values found in all sampling times (S1, S2, and S3).

**Figure 1:**
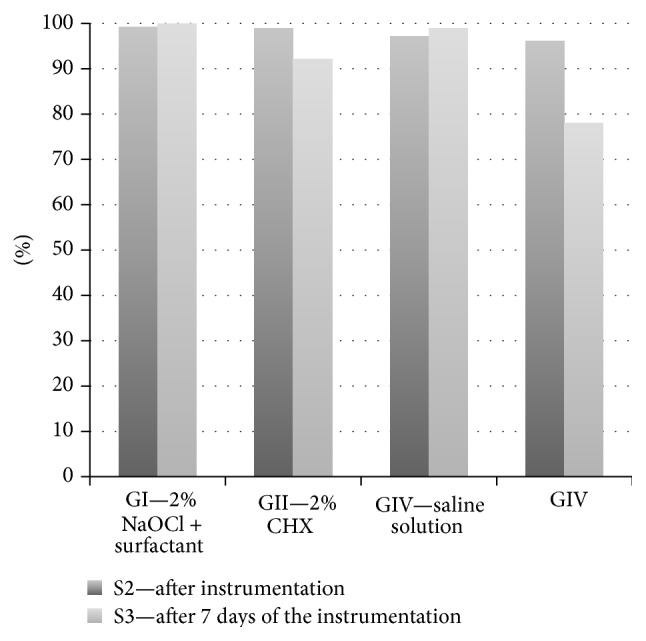
Descriptive analysis of the reduction percentages in the first and second samplings (S2 and S3) in relation to the initial sampling (S1).

In the baseline samples (S1), LAL assay indicated that endotoxins were found in all root canals (40/40), with median unit (EU/mL) ranging from 707 to 124.000. At S2, a higher percentage of endotoxin reduction was found in GI (2% NaOCl + surfactant) compared to GII (2% CHX) and GIII (2.5% NaOCl) (*p* < 0.05). Moreover, at S3, 2% NaOCl + surfactant was the most effective solution against endotoxins (Figure 1). At S2, the percentages of endotoxin reduction found in GI, GII, and GIII were, respectively, 99.30%, 98.90%, and 97.18%, whereas GIV had 96.11%. At S3, the percentages of endotoxin reduction were 100%, 92.14%, 99.00%, and 78.04%, respectively, for GI, GII, GIII, and GIV. The descriptive analysis of the reduction percentages in the first and second samplings (S2 and S3) in relation to the initial sampling (S1) is presented in Figure 1.

## 4. Discussion

In the present study, the association of 2% NaOCl with surfactant resulted in lower bacterial growth and greater endotoxin reduction in comparison with 2.5% sodium hypochlorite and 2% chlorhexidine.

It is known that the antimicrobial effectiveness of NaOCl in eradicating microorganisms is increased by its direct contact and mechanical entrainment of volatile gases released during this contact, leading to the removal of microorganisms [18]. The wettability of the irrigant plays a major role in obtaining a suitable contact time between NaOCl and root canal dentinal walls. In fact, wettability is correlated with surface tension [18] of ideal surfaces (i.e., chemically homogeneous, flat, nonreactive, undeformable, and not swollen by the wetting liquid) and with the surface properties of dentin [19].

Moreover, as the surfactant is a detergent, it possesses emulsifying properties. These properties facilitate the removal of debris from the dentin surface by maintaining them on suspension, which increases dentin wettability and facilitates instrumentation [21].

The physicochemical properties of irrigants characterize their clinical behavior during instrumentation. One of these properties is the optimal wetting. The tendency of a liquid to spread on the solid surface depends on the formation of contact angle [26]. A surface with lower contact angle (i.e., higher surface free energy) presents high wettability; that is, in one solid with high surface free energy, the auxiliary chemical substance spreads and interacts better with this surface, forming a low contact angle.

The foam formation during instrumentation with NaOCl + surfactant might have contributed to a better reduction of bacterial load, particularly against* E. faecalis*, as well as to a significant improvement in the removal of endotoxins. Also, the foam formation might have aided the separation between dentin walls and adhered debris, working as insulation between microorganisms, smear layer, and dentin walls by keeping debris in suspension and facilitating their removal [27].

Currently, 2% sodium hypochlorite with surfactant has shown the highest detoxifying activity against endotoxins compared to all other substances tested. Moreover, no statically significant difference was found when comparing 2.5% NaOCl to 2% chlorhexidine. The effectiveness of NaOCl in reducing endotoxins in root canal infection had been previously demonstrated [28]. This can be explained due to the properties of the surfactant, also known as detergent. The surfactant properties seem to increase the diffusion capacity of NaOCl into dentin tubules and root canal system, thus allowing its action against microorganisms deeply positioned into the dentin mass [27].

No auxiliary chemical substance tested was effective in eliminating endotoxins from the root canals. After 7 days of root canal instrumentation, a higher number of root canals that remained free of endotoxins were found in the group of 2% sodium hypochlorite + surfactant compared to all other groups tested. The limited effectiveness of root canal instrumentation in eliminating endotoxins from root canals, as demonstrated in the present study and elsewhere [6, 9], elucidates the importance of the use of intracanal medication in order to achieve an optimal disinfection.

Although 2% CHX solution has demonstrated a high antimicrobial activity, it showed the lowest efficacy against endotoxins compared to all other substances tested. Its high antimicrobial activity might be explained by its positively charged molecules. The electrostatic interaction between CHX and cellular walls increases the bacterial permeability, allowing CHX to diffuse into bacterial cytoplasm and then causing death [29]. Regardless of the low detoxifying activity of CHX against endotoxins, it is still a good alternative in those teeth with large foramen and with incomplete root formation because of its low toxicity [10].

Among the species tested,* E. faecalis* was the only microorganism recovered from root canals immediately after instrumentation and after 7 days of root canal instrumentation, which supports its capacity to survive to root canal procedures. Previous study had reported that chlorhexidine exhibits residual antimicrobial activity [30, 31]. However, in the present study,* E. faecalis *was recovered from root canals after 7 days of biomechanical preparation. The low effectiveness of chlorhexidine in eliminating* E. faecalis* from root canals might be related to its limited ability in removing the smear layer formed during the biomechanical preparation [32], which can obliterate dentinal tubules and root canal system.

## 5. Conclusion

All irrigants tested were effective in reducing microorganisms and endotoxins from infected root canals. Moreover, 2% NaOCl + surfactant was the most effective irrigant tested against endotoxins and regrowth of microorganisms.

## Figures and Tables

**Table 1:** Distribution of the median colony-forming unit (CFU/mL) and median percentage of bacterial load reduction found at all different sampling times (S1, S2, and S3).

Groups	Microorganisms	Baseline (S1)		After instrumentation (S2)			After 7 days (S3)		
		CFU/mL	Positive culture	CFU/mL	Negative culture	Median % valued reduction (S1-S2)	CFU/mL	Negative culture	Median % valued reduction (S1–S3)
GI NaOCl + surfactant	*E*. *faecalis *	2.0 × 10^7^	10/10	0	9/10	100 (50.33–100)	0	10/10	100
	*C*. *albicans *	2.55 × 10^5^	10/10	0	9/10	100 (48.53–100)	0	10/10	100
	*E*. *coli *	3.12 × 10^5^	10/10	0	10/10	100	0	10/10	100

GII 2% CHX solution	*E*. *faecalis *	9.25 × 10^7^	10/10	0	10/10	100	0	9/10	100 (62.91–100)
	*C*. *albicans *	3.06 × 10^5^	10/10	0	10/10	100	0	10/10	100
	*E*. *coli *	3.36 × 10^5^	10/10	0	10/10	100	0	10/10	100

GIII 2.5% NaOCl	*E*. *faecalis *	2.15 × 10^7^	10/10	0	10/10	100	1.0 × 10^2^	7/10	72.6 (55.58–100)^*∗*^
	*C*. *albicans *	1.10 × 10^5^	10/10	0	10/10	100	0	10/10	100
	*E*. *coli *	3.19 × 10^5^	10/10	0	10/10	100	0	10/10	100

GIV Control group	*E*. *faecalis *	1.84 × 10^7^	10/10	5.0 × 10^1^	0/10	33 (29.62–42.69)^*∗*^	1.0 × 10^3^	0/10	30.88 (26.47–36.13)^*∗*^
	*C*. *albicans *	1.06 × 10^5^	10/10	8.96 × 10^4^	1/10	80.63 (26.83–100)^*∗*^	1.40 × 10^5^	1/10	78.10 (29–100)^*∗*^
	*E*. *coli *	3.52 × 10^5^	10/10	4.48 × 10^2^	0/10	36.39 (21.59–42.69)^*∗*^	4.32 × 10^4^	0/10	38.53 (19.51–47.93)^*∗*^

^*∗*^Statistical differences (*p* < 0.05).
